# Discovering Speed Changes of Vehicles from Audio Data

**DOI:** 10.3390/s19143067

**Published:** 2019-07-11

**Authors:** Elżbieta Kubera, Alicja Wieczorkowska, Andrzej Kuranc, Tomasz Słowik

**Affiliations:** 1 Department of Applied Mathematics and Computer Science, University of Life Sciences in Lublin, 20-950 Lublin, Poland; 2 Department of Multimedia, Polish-Japanese Academy of Information Technology, 02-008 Warsaw, Poland; 3 Department of Energetics and Transportation, University of Life Sciences in Lublin, 20-950 Lublin, Poland

**Keywords:** speed changes detection, road traffic safety, audio signal analysis

## Abstract

In this paper, we focus on detection of speed changes from audio data, representing recordings of cars passing a microphone placed near the road. The goal of this work is to observe the behavior of drivers near control points, in order to check whether their driving is safe both when approaching the speed camera and after passing it. The audio data were recorded in controlled conditions, and they are publicly available for downloading. They represent one of three classes: car accelerating, decelerating, or maintaining constant speed. We used SVM, random forests, and artificial neural networks as classifiers, as well as the time series based approach. We also tested several approaches to audio data representation, namely: average values of basic audio features within the analyzed time segment, parametric description of the time evolution of these features, and parametric description of curves (lines) in the spectrogram. Additionally, the combinations of these representations were used in classification experiments. As a final step, we constructed an ensemble classifier, consisting of the best models. The proposed solution achieved an accuracy of almost 95%, without mistaking acceleration with deceleration, and very rare mistakes between stable speed and speed changes. The outcomes of this work can become a basis for campaigns aiming at improving traffic safety.

## 1. Introduction

Although some drivers will not admit it, driving at excessive speed increases the difficulty of carrying out the tasks they face while driving. They have less time to properly evaluate the traffic situation and respond accordingly. Also, the vehicle’s braking distance increases with the increasing speed, and the other road users have less time to avoid an accident. Excessive speed, i.e., unmatched to road and traffic conditions, is the cause of many road accidents and their consequences, as evidenced in numerous works on the subject [1,2,3,4,5,6,7,8]. To improve this situation, careful investigation and monitoring of trends in road safety is needed. To this end, a network of road safety observatories has been build. European road safety data and knowledge have been integrated and made publicly available on the Internet [8,9]. They can be used to devise new initiatives, take appropriate policy decisions, and implement corrective measures if necessary [4,9]. Speeding offences, along with drink driving and not wearing of seat belts in cars and helmets on motorcycles, are the main causes of crashes resulting in serious and fatal injuries [4].

The Power Model, developed in the 1980s by a Swedish road safety researcher, G. Nilsson, defines the relationship between speed and road safety via power functions, rather than linear ones [10]. Six equations represent the model, with varying exponent values. The equations describe: fatalities, fatal and serious injuries, all injured road users, fatal accidents, accidents involving fatal or serious injury, and all injury accidents. For instance, the Power Model for fatal accidents is expressed by the following equation (assuming that the average driving speed changes and all other factors remain unchanged):

$$\frac{F_{acc-before}}{F_{acc-after}}={\left(\frac{S_{before}}{S_{after}}\right)}^{4}$$

where: $F_{acc-before}$ is the number of fatal accidents before speed change, $F_{acc-after}$ is the number of fatal accidents after speed change, $S_{before}$ is the speed before change, $S_{after}$ is the speed after change. An exponent of 2 is proposed for all injury accidents, 3 for fatal and serious injuries, between 2–4 for all injured road users, etc. Therefore, a 1% change in average driving speed leads to a 4% increase in fatal accidents, a 3% increase in fatal and serious injuries, a 2% increase in all injury accidents, etc. These relationships show that speed is a very important factor influencing road accidents.

The connection between speed and accident risk is widely accepted by traffic safety experts; speed lower than the average increases crashes only marginally [11,12]. Moreover, the reason the drivers are driving slower could be in response to other risk-increasing condition (fatigue, poor vision, major distraction, vehicle problems) [13].

In Poland, road accidents generate enormous economic and social costs, and their main cause is excess speed, not adjusted to weather and road conditions, visibility, and traffic [14]. Also, many drivers accept speeding as a standard. Additionally, some drivers use roads as racing tracks. Many drivers think that the road accidents are not only caused by driving too fast, but also by too slow driving that causes dangerous behavior of other drivers (e.g., overtaking on a solid line) [15]. Obviously, slower-moving vehicles, such as recreational vehicles, should avoid using the left lane, as this reduces the efficiency of the highway system, and causes driver frustration, also observed in other countries [16]. This results in aggressive and erratic driving behavior which really is unsafe for everyone.

The widespread acceptance of speeding on Polish roads and high tolerance for reckless driving is worrying. The drivers typically exceed the speed limit by 20–30 km/h. If not in traffic jams, they drive at an average speed of 70–80 km/h in urban areas (i.e., with 50 km/h speed limit), and 110–120 km/h in rural areas with 90 km/h speed limit [17].

The choice of driving speed depends on the motivation of the driver, his or her attitude, as well as risk perception and acceptance. Driving speed is also influenced by the estimated probability of police control, the technical condition and parameters of the driven vehicle, and the characteristics of the road and its surroundings. However, the impact of speeding on the decrease of the travel time is overestimated, and the travel time increase in the case of slower driving is overestimated too [15].

Driving behavior has been already investigated worldwide. Quantitative estimation of various elements of driving can be based, e.g., on information extracted from audio and video data [18,19], global positioning system (GPS) data [20], or Controller Area Network (CAN) data, i.e., the accelerator opening rate, brake pedal pressure, and steering wheel angle [21,22]. Mobile devices are applied to collect such data as well [23,24]. Driver behavior models are also built, and used in simulators and in vehicles; they can warn drivers of adverse speeding (warning systems), or limit speed by using mechanical systems (mandatory systems) and techniques such as throttle control and braking, or combine these two methods. The mandatory systems, however, are extremely negatively perceived by drivers [25].

Based on the observation of over 6000 drivers passing by 50 speed cameras located all over Poland, the undesirable behavior of drivers can be observed. It is often caused by the use of a car navigation application, combining the navigation system with a video recorder, and a warning system, which warns the driver of a police radar speed trap [26]. Drivers brake violently about 450 m ahead of the speed camera, and they vigorously accelerate just after passing by the camera. They drive at a safe speed of 50 km/h (if it is the speed limit) only about 100 m ahead and 100 m behind the speed camera, whereas about 1 km ahead of the camera they drive at 90–95 km/h, i.e., almost twice the legal speed limit. In extreme cases, the average speed after slowing down in front of the speed camera was about half of the speed limit. This situation was observed in Kłoda in Greater Poland voivodship (Rydzyna municipality, speed limit—70 km/h), where the drivers passed a speed camera with an average speed of 36 km/h, and in Radomsko (ódź voivodship), where the measured speed was 25 km/h less than the speed limit. Another example comes from the vicinity of a speed camera in Kaźmierzów (Lower Silesia voivodship). Here the drivers reduced speed from 94 to 53 km/h for about 300 m, passing by the camera at a speed of 15 km/h lower than the speed limit of 70 km/h, thus decreasing traffic flow [27].

The driving pattern of braking and accelerating is observed in other countries as well [28,29]; it is called the kangaroo effect, or kangaroo driving. Still, even though speed cameras can cause a kangaroo effect, they reduce total crash numbers by about 20%. However, the effect declines with increasing distance from the speed camera location [28].

The perturbation of traffic flow because of decelerating in kangaroo driving causes the drivers to try to make up for lost time, and they do this by speeding. Such a situation creates a vicious cycle, reinforced by the negative behavior of road users [4,5,8].

It should also be noted that the perturbation of traffic flow causes traffic congestion. This is documented by TomTom [30], a company specializing in navigation and mapping products, and also measuring and publishing traffic index, describing congestion worldwide. Taking Congestion Index 2018 [30] and Warsaw as example, the commuting time at rush hours almost doubled. Additional time spent in the car during peak hours in the evening was 25 min per 30 min trip (without traffic jams). This situation causes an irresistible urge to make up for the lost time and subsequently pushes drivers to exceed speed limits.

The importance of driving speed and its relation to road safety explains the need of monitoring the speed of vehicles on roadways. Many research studies [1,15,17] indicate that the traffic safety can be improved by implementing speed management. The speed management strategies include surveillance and legal measures, information campaigns and advanced technologies, as well as managing road infrastructure. The purpose of speed management is to achieve traffic flow with safe speed, adapted to road conditions.

Speed cameras take speed measurements based on an integrated Doppler or laser radar, and since they register the picture of the vehicle, licence plate numbers can also be recognized using automatic licence-plate recognition (ALPR) [31]. In early systems, ALPR recognized about 70% of number plates. Under perfect conditions, ALPR systems nowadays identify correctly over 90% of licence plates [31]. The speed can be also measured through frame-by-frame image analysis, i.e., time-lapse analysis of the movement of the licence plate in the measurement field. Other measurement methods used in vehicle traffic research include various sensors. Two inductive-loop detectors which sense the presence of a vehicle can be applied to measure speed. However, these loops should be spaced relatively far apart, in order to minimize the differences between various vehicle types. Magnetic sensors can be applied instead of loops, and they are less susceptible to inclement weather. Such sensors can be used in conjunction with the pneumatic detectors, which are pressure-sensitive detectors, using pneumatic tubes. However, pneumatic sensors are costly to install, and capable of detecting the presence of vehicles in motion only [32,33]. Vehicle traffic is also monitored on railways, using rail wheel proximity sensors [34].

Average speed of vehicles over routes between distant sites is also sometimes measured. It is calculated by dividing the known distance between the observation sites by the time the vehicle needed to travel between these spots.

## 2. Use of Audio Data in Automotive Research

The measurement methods mentioned above have advantages and disadvantages. Drawbacks may include high installation cost, detecting only passing (in motion) vehicles, or susceptibility to inclement weather. Additionally, these systems output only one value, representing instantaneous or average speed value, and speed changes are not measured. Therefore, a microphone-based system that determines whether a vehicle slows down, moves at a constant speed, or accelerates, seems to be quite promising, and in conjunction with an ALPR system could become a complete preventive system for evaluating drivers behavior on the road. In particular, such a system could be used to assess the behavior of drivers notorious for traffic violations, who are trying to avoid penalties.

The advantages of the microphone-based system include the independence of its operation from the lighting conditions at the site of measurement, and the independence from satellite navigation systems (e.g., in tunnels, under heavy clouds, etc.). Another advantage is the ability to measure the speed and its changes both before and after the measurement point. Therefore, such a system will allow analyzing the behavior of drivers passing by the speed enforcement system. In addition to vehicle speed measurement, acoustic systems can also be used to measure vehicle traffic density and to evaluate whether gears in manual transmission systems are optimally used [35,36]. Acoustic signals have been also successfully used to detect and classify hazardous events on roads such as car crashes and tire skidding [37].

Audio data have been used in automotive research in various applications, including estimating vehicle speed and speed changes [36,38], or to assess traffic density [39]. In [36], the sound of a moving vehicle was classified into discretized speed intervals of 1, 2, 3, 5, 7 and 10 km/h, with classification results over 90% even for smaller intervals; gear position was also classified based on audio data, but the results ranged between 55–70%. In [38], audio data of passing vehicles were classified into 3 classes: acceleration, deceleration, and constant speed, with various accuracy, depending on data representation and classifier, exceeding 95% in best cases (for a limited and rather homogenous data set). Audio data are usually down-sampled and low-pass filtered, as high frequencies are not needed in this research.

Audio data were also applied in research on automatic failure classification [40,41,42]. In [42], engine misfire detection was investigated, based on audio data acquired via mobile phone. The accuracy in this research reached 99%. Engine fault diagnosis using audio data was also investigated in other works, see [43,44] for example. In [40], acoustic signals were applied to detect multiple faults without opening the engine. The authors managed to detect air filter fault, spark plug fault, piston ring fault, insufficient lubricants faults, and excessive lubricants fault, reaching 94% accuracy for two stroke engine. In [41], the investigated fault was within the ignition system of the engines, and classification results of about 80% were reported.

Additionally, audio data were applied to vehicle detection and classification into categories, i.e., car, van, truck, bus, motorcycle etc. [45,46,47,48,49]; in [50], seismic data were additionally used. In [45], 93.74% correctness was obtained in classification of vehicles into three classes: car, motorcycle, and truck. In [46], 87% correctness was obtained for low speed, and 83% for higher speed, for another three classes, namely car, truck, and van. The accuracy of 67% was obtained in [47] for 4 classes: heavy vehicles, medium vehicles, light vehicles, and horns. In [48] 80% of correct classification was obtained also for 4 classes: bus, car, motor, and truck. In other research [49], where seven vehicle classes were examined, namely bus, small truck (without trailer), big truck (tractor unit with semitrailer), van, motorcycle (excluding scooters), car, and tractor, audio data were also applied for vehicle and engine classification. The results reached 74.4–99% accuracy. For engine classification according to types of fuel used, i.e., gasoline and Diesel, the accuracy of 97.7% was obtained for dyno bench test recordings.

### 2.1. Parametrization and Classification of Audio Data

Audio data are usually parameterized before classification. Typical audio features (often designed for music analysis, and implemented in publicly available libraries) include [51,52,53,54]:

- time domain parameters: Zero Crossing Rate (the rate of sign-changes of the signal in a particular frame), or Zero Crossing Rising (the number of times when the audio signal rises to 0 and becomes positive);
- Energy;
- spectral features: Spectral Centroid (the gravity center of the spectrum), Spectral Spread (the second central moment of the spectrum), RollOff (the frequency below which 85–90% of the spectrum is concentrated; the threshold value depends on implementation), Spectral Flux (the squared difference between the normalized magnitudes of the spectra of two successive frames);
- Mel Frequency Cepstral Coefficients (MFCC), a cepstral representation where the frequency bands are distributed according to the mel-scale;
- features designed to describe music: Pitch, Chroma Vector (12-element vector, with elements representing the spectral energy of the 12 pitch classes in equal temperament); Harmonic Spectral Centroid and Harmonic Spectral Flux.

Sound classification is often performed based on a feature vector, using features mentioned above, and also features designed for specific research purposes. However, spectrogram-based representations are also used [55,56,57,58,59]. These representations are often used in classification based on artificial neural networks. In [55,59], deep learning approaches were applied, taking mel-spectrograms as input to convolutional neural networks. Other classifiers were also applied, including support vector machines (SVM) and random forests [56,58], or hidden Markov models (HMM), often used on audio data [60]. Obviously, acoustical data after parameterization can be classified using any classification algorithms, e.g., k-nearest neighbors [60].

In this paper, we propose to detect changes of speed of vehicles on road, based on audio data. To start with, we recorded audio data representing single vehicles in controlled conditions, to assure proper representation of accelerating, decelerating, and maintaining stable speed. Next, audio data were parameterized. Several representations were tested, based on various audio features and spectrogram data. Next, classification was performed on the parameterized data, to test the feasibility of the proposed approach. We experimented with random forests, neural networks, and support vector machines as classifiers. Since our data represent changes in time, we also applied a time series based method. A classifier ensemble was used for final classification. The obtained results are presented and discussed in the paper.

## 3. Materials and Methods

**Figure 1:**
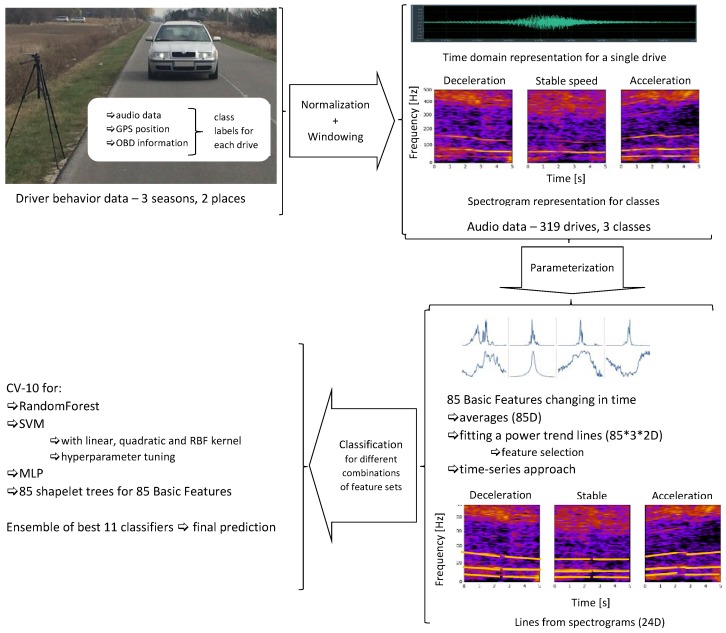
Our proposed methodology of research on evaluating speed changes from audio data.

In this work, we propose a research methodology based on evaluating speed changes from audio data, see Figure 1, as this methodology can help collecting information about the behavior of drivers around speed cameras, without the need of acquiring data from sensors installed in these vehicles. First of all, we recorded audio data representing single vehicles in controlled conditions. We acquired on-road recordings of single drives of single cars, driven by the authors, to assure proper controlled accelerating, decelerating, and maintaining stable speed. Each drive in our audio data consisted of 10 s of data, centered at the moment of passing the microphone. We also acquired GPS (Global Positioning System) and OBD (On-Board Diagnostics) data if available. The reason was to verify the results of our calculations and to facilitate the preparation of data for experiments. GPS data were used to find the approximate time of passing the microphone. The exact moment of passing the microphone was found as the maximum of the signal envelope within the range ±2 s of the point indicated by GPS data (exact to 1 s).

Next, the acquired audio data were parameterized. We experimented with various representations, based on audio features and spectrogram data. Afterwards, classification was performed on our data, using classifiers already applied in research on audio data classification. We selected SVM, random forests, and artificial neural networks to test the feasibility of the proposed approach. We also applied a time series based method. Fast shapelet algorithm was used to determine so called shapelets, see Section 3.3. Each shapelet represents a shape specific for the evolution of a feature in time, and data classification is based on decision trees, built for each feature separately. We decided to use a forest of such trees as a shapelet-based classifier. Finally, a classifier ensemble consisting of best performing classifiers was used for final classification of the investigated audio data.

### 3.1. Audio Data

In order to evaluate the proposed approach, we acquired audio data in controlled conditions. Mc Crypt DR3 Linear PCM Recorder, with 2 integrated high-quality microphones, was used to record audio data, 48 kHz/24 bit, stereo. The data were recorded on side roads, as we aimed to record one vehicle at a time and avoid interfering vehicles, if possible. The cars used in the recordings were driven by one of the authors, T. Słowik or A. Kuranc. We recorded our on-road data in three seasons:

1. in the summer, on August 2nd 2016, in Ciecierzyn, Lublin voivodship, Poland,
2. in the winter, on January 16th 2017, in Lubartów, Lublin voivodship, Poland,
3. in the spring, on March 31st 2017 and on April 5th 2017, in Lubartów, Lublin voivodship, Poland.

All these data were used together as one data set. Each data item represents one drive, which can be classified into one of 3 classes: accelerating, decelerating, or maintaining stable speed (as much as possible, although small fluctuations are unavoidable). Altogether, we recorded 114 drives representing deceleration, 94 drives representing stable speed, and 111 drives representing acceleration. A road segment of about 300 m was used for each drive, and deceleration/acceleration was performed from about 60 m before up to 60 m after the passing the microphone.

Data

The recorded audio data are available at kzmi.up.lublin.pl/~ekubera/drives.zip. In this archive the data are divided into three folders: Ciecierzyn, Lubartow_winter, and Lubartow_spring. In each folder, there are three subfolders: 0, containing deceleration samples, 1 containing stable speed samples, and 2 containing acceleration samples. Audio data are represented in .wav or in .au format.

#### 3.1.1. Summer Recordings

**Figure 2:**
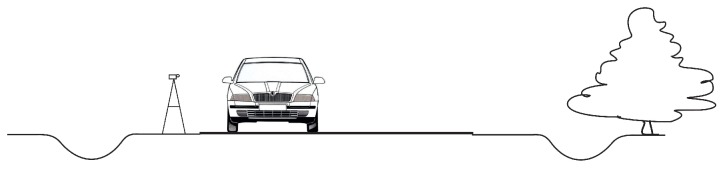
The position of the audio recorder with stereo microphones during on-road recordings in Ciecierzyn in the summer.

These data were recorded for two cars with Diesel engine (Toyota Corolla Verso and Skoda Octavia), and one car with gasoline engine (Renault Espace). We recorded 20 drives, i.e., two drives for each car per class, with additional two drives of Skoda. The drivers accelerated from 50 to 70 km/h, decelerated from 70 to 50 km/h, or maintained stable speed of 50 km/h, plus two drives at 70 km/h for Skoda. The recordings were performed on a sunny day (weekday). The audio recorder with stereo microphones was positioned 1.5 m above the surface and as close to the road as possible, see Figure 2. This part of the roadway was situated in a mild basin, broad enough to assure that the cars were not driving uphill nor downhill. The data were recorded in the morning from around 10 a.m. to noon.

#### 3.1.2. Winter Recordings

**Figure 3:**
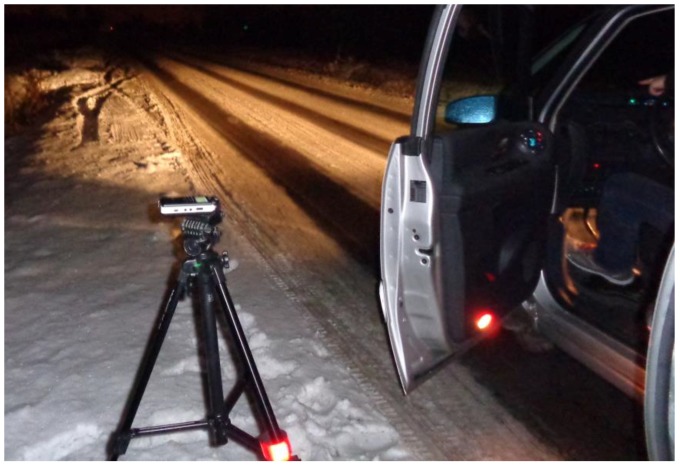
The road and the position of the audio recorder with built-in stereo microphones during on-road recordings in Lubartów in the winter.

These recordings were made on a road in the outskirts of a small town in Poland [58]. There was snow on the road, but not on the area below the tires, see Figure 3. The car used in the recordings was a Renault Espace IV (2007). The recorded data included 84 drives, representing 28 drives with acceleration from 50 to 70 km/h, 28 drives with stable speed, 50 km/h, and 28 drives with deceleration from 70 to 50 km/h. The car was equipped with manual transmission, and all drives were recorded without changing gear and without applying brakes (engine braking only). The data were recorded around 6–8.30 p.m.

#### 3.1.3. Spring Recordings

**Figure 4:**
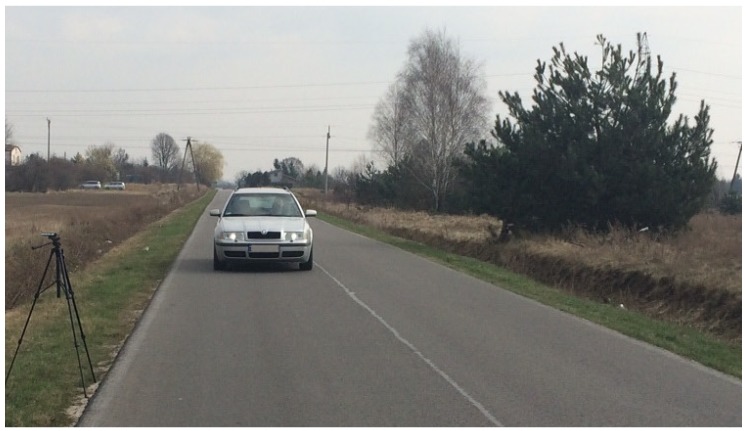
The road and the position of the audio recorder with stereo microphones during on-road recordings in Lubartów in the spring.

These recordings were made in early spring, on 31 March 2017, in the morning (between 8.30 and 11.15 a.m.) and on 5 April 2017, also in the morning (between 8.30 and 11.30 a.m.). The weather was windy on 31 March and we had good weather on 5 April. The initial part of the recordings taken on 31 March has wind gusts, and later a windscreen was applied to the microphones. The wind gusts did not affect the audio data of the cars passing by, so we used these data in further works. We chose the same road as in the winter, see Figure 4.

On 31 March we recorded Renault Espace III, with a gasoline engine, and Skoda Octavia, with a Diesel engine. On 5 April we recorded Skoda Octavia again, Smart ForFour with a gasoline engine, and Renault Espace IV with a gasoline engine. All vehicles were equipped with a manual transmission. We recorded:

- 77 drives for acceleration, from 50 to 70 km/h, and from 50 to 80 km/h for Skoda Octavia;
- 58 drives for stable speed, at 60, 70, and 80 km/h; the speed limit in this area is 90 km/h;
- 80 drives for deceleration, with the following speed ranges: 80–40 km/h, 80–50 km/h, and 70–40 km/h; deceleration included applying brakes in these recordings, as we wanted to obtain data corresponding to more intensive deceleration.

### 3.2. Data Parametrization

**Figure 5:**
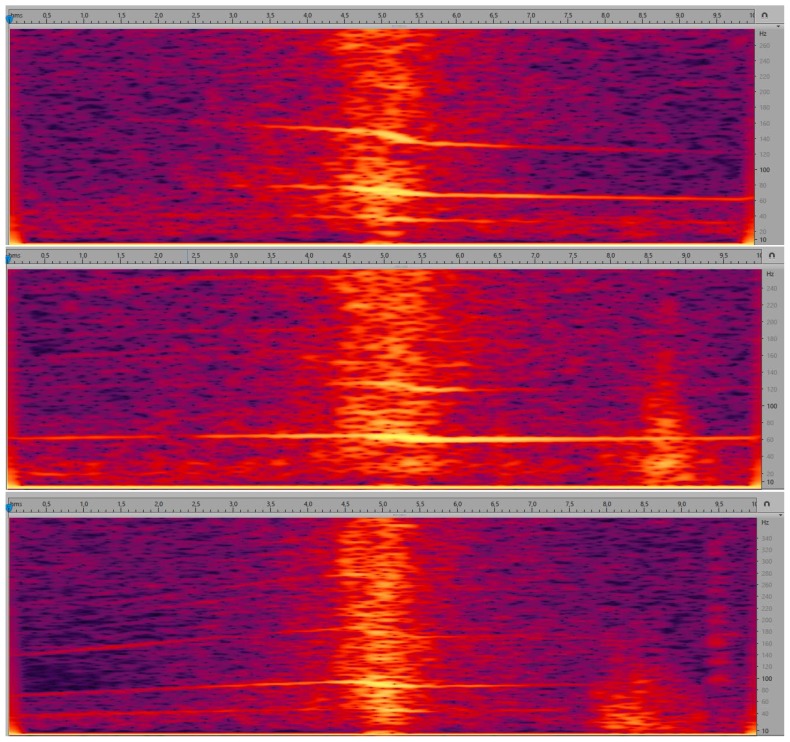
Spectrograms for deceleration (**upper graph**), stable speed (**middle graph**), and acceleration (**lower graph**). Horizontal axis represents time, vertical axis represents frequency. Higher luminance (brighter color) corresponds to higher amplitude. Graphs obtained from Adobe Audition [63].

In this work, we decided to test several approaches to audio data representation, namely:

- feature vector, consisting of 85 features calculated for consecutive frames (with 2/3 frame overlap) of the entire parameterized audio segment and then averaged;
- parametric description of the evolution of 85 basic features in time, observed within the analyzed audio segment on frame-by-frame basis; these trends were also approximated separately for the time segments before and after passing the microphone;
- time-series based approach, where the time evolution of our 85 features was represented through so-called shapelets, i.e., maximally representative subsequences for each class in classification [61,62];
- parametric description of curves (lines) in the spectrogram, calculated separately for the time segment before approaching the microphone and for the time segment after passing the microphone. Spectrogram shows the evolution of spectrum over time, i.e., horizontal axis represents time, vertical axis represents frequency, and spectral coefficients are encoded using a selected color scale. Exemplary spectrograms for our audio data are shown in Figure 5. As we can see, lines can be observed in these spectrograms, but very few of them are pronounced.

These representations were next used in classification. Since we had stereo data, we decided to use them separately. Namely, if the classifier was trained on the left channel, it was also tested on the left channel data, to avoid over-optimistic results if the data in both channels were almost identical.

#### 3.2.1. 85 Basic Features

The starting point in our work with the recorded audio data is parametrization yielding a feature vector consisting of the following 85 parameters:

- *Zero Crossings Rising* (ZCR);
- *Audio Spectrum Envelope* (SE) $SE_{0},\ldots SE_{32}$ calculated as sums of the power spectrum coefficients within logarithmically spaced frequency bands [53];
- *SumSE*—sum of the spectrum envelope values;
- *MaxSE, IndMaxSE*—value and index of the SE maximum;
- *Audio Spectrum Flatness*, *Flat1*, …, Flat25—a vector describing the flatness property of the power spectrum [53], i.e., the deviation of the signal’s power spectrum from a flat shape; flat spectrum corresponds to noises, otherwise the spectrum has tonal components;
- *MFCC*—13 coefficients. The cepstrum is calculated as the logarithm of the mel-scaled magnitudes of the spectral coefficients, submitted to DCT (discrete cosine transform); the mel scale reflects the properties of the human perception of frequency; 24 mel filters were applied, and we took the first 12 coefficients. The thirteenth coefficient is the 0-order coefficient of MFCC, corresponding to the logarithm of the energy;
- *F0_ACor*—fundamental frequency, calculated from the autocorrelation function; this frequency changes with the speed change;
- *Energy*—energy of the entire spectrum;
- *EnAb4kHz*—proportion of the spectral energy above 4 kHz to *Energy*;
- *Audio Spectrum Centroid* (SC) [53];
- *Audio Spectrum Spread* (SS); [53];
- *RollOff*—the frequency below which 85% of the accumulated magnitudes of the spectrum is concentrated,
- *BW10dB, BW20dB, BW30dB*—bandwidth of the frequency band comprising the power spectrum maximum and the level drop by 10, 20 and 30 dB, respectively, towards both lower and upper frequencies;
- *Flux*—the comparison of the power spectrum for a given frame and the previous one, calculated as the sum of squared differences between the magnitudes of the DFT points in the given frame and their corresponding values in the preceding frame.

These features were calculated for consecutive 170 ms frames (with 57 ms hop size, i.e., with 2/3 frame overlap) of each 10 s audio segment, and feature vector consists of the values averaged over the segment. For 48 kHz sampling rate, 170 ms of data consists of 0.170·48,000=8160 audio samples.

In order to calculate the spectrum (and then the spectrogram), the segmented data were first normalized to 0 mean. Hamming windowing was applied before calculating spectrum, i.e., the audio signal samples x[n] were multiplied by

w[n]=0.54−0.46cos(2πn/N),n=0,…,8159,N=8160.

Next, the spectrum was calculated through Fast Fourier Transform (FFT) for our 170 ms frames (8160 samples), zero-padded to 8192 samples; the FFT implementation requires the frame length to be the power of 2.

The calculated features were normalized to [0, 1] before classification. The vector of 85 basic features consists of the average values of each feature in all analyzed audio frames.

#### 3.2.2. Trends

**Figure 6:**
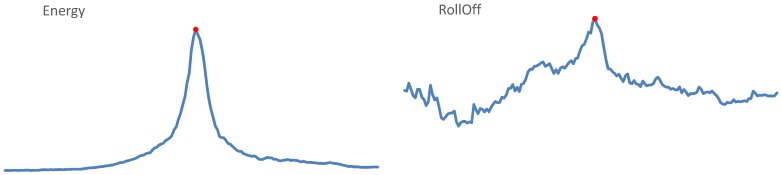
Evolution of exemplary features (Energy and RollOff) in time through the entire 10 s segment. The central point is marked in red.

This representation of our audio data is based on observing how feature values for each of 85 basic features change in time, within the observed 10 s audio segment. The trends of time evolution of our features were calculated separately for 5 s before and for 5 s after passing the microphone. Exemplary evolutions of features in time are shown in Figure 6. As we can see, feature values may increase smoothly before the central point, representing passing the microphone, and decrease afterwards, or vary in time in less clear order. We decided to approximate the trends of evolution using the power trend, i.e.,

$$y=b\cdot x^{a}$$

where *a*, *b*—coefficients calculated through the least squares method. Additionally, the coefficient of determination $R^{2}$ was added. Therefore, we obtained 3 coefficients for the first 5 s and 3 coefficients for the second 5 s. As a result, for each of the 85 features we obtained 6 values representing the trends of these features in time. Therefore, the feature vector representing trends consisted of 6·85=510 features.

#### 3.2.3. Time Series-Based Approach

Shapelets were extracted for the data corresponding to the evolution of the 85 basic feature values in time, for each feature separately. A shapelet is a shape representative for a given decision class. Shapelets are extracted from the training data as segments of the data of the length shorter than the full series. We based this representation on fast shapelet algorithm, which requires fixing the length of shapelets [62]. In our case, we looked for the best matching shapelet of length 10, …, 170, with the step of 10 points.

#### 3.2.4. Lines

In this representation, we parameterized the lines of the highest energy up to 500 Hz in the spectrogram. Several methods of calculating lines were tested, as finding pronounced, continuous lines spanning through the entire spectrogram required careful calculations. In all cases, we approximate by means of linear functions (using the method of least squares) three lines in 5 s before and three lines in 5 s after the moment of passing the microphone. Each line is represented through the intercept, gradient, and quotient of the ending and starting frequencies, i.e., three values per line. Additionally, we added the logarithm of the intercept (base 2) to these features. Therefore, this representation consists of 24 values, 12 for three lines before and 12 for three lines after passing the microphone. The following methods were applied to calculate these values:

Method 1 In the 5 s when the vehicle is approaching the microphone, we look for the three lines of the highest energy, spanning slantwise in the spectrogram (based on 8192-element spectrum), as follows [58]:
  - For each frame, we look for three candidates for the lines. We analyze the following ranges: 50–100 Hz, 100–200 Hz, 200–300 Hz, 300–400 Hz, and 400–500 Hz. Frequencies most often indicated as local maxima in these ranges are returned as candidates to start lines. For the selected three candidates, three continuous paths in the spectrogram are traced starting from the moment of passing the microphone (towards the decrease of energy). We traced the paths through following local maxima, i.e., in the next frame we considered the average of the last three frequencies (if available) already added to the line and its two neighbors. Next, for each path we find a line, which best approximates this path, by means of linear approximation.
  - Similarly, we look for three lines in the 5 s segment after passing the microphone.
  This solution has several drawbacks, namely we can lose the path, or the algorithm may follow the path even if the line is no longer visible in the spectrogram, and each point influences the line with the same weight.
Method 2 In this method we decided to down-sample audio data to 1 kHz sampling rate; low-pass filter was applied here (averaging). We aimed at high-resolution spectrogram, so we decided to perform zero-padding of our 170 ms long frames to 1024 samples, thus achieving spectral bins of 1 Hz. The procedure of calculating lines remained the same.
Method 3 In this case we calculated the logarithm of the energy (base 10), and frequency was also expressed in logarithmic scale (base 2). On a logarithmic scale lines are parallel on the spectrogram.
Method 4 Since we expected harmonic lines (i.e., for frequencies representing multiples of the lowest, fundamental frequency) and the obtained lines were often situated very close to each other, we decided to simplify the procedure of searching for candidates. The candidate from 50–150 Hz was considered to represent the first line, with maximal value of energy in the center of our 10 s segment. Additionally, we corrected octave errors, i.e., erroneous indication of the double of the fundamental frequency.
Method 5 Weighted linear regression. The linear regression was weighted with $\left(1-E_{N}\right)$, where $E_{N}$ is the normalized energy of each point, on a logarithmic scale.
Method 6 Modification of method no. 4 by changing the starting moment of tracing lines. Namely, the tracing starts from the time moment when the energy is 10 dB less than in the loudest moment (center, passing the microphone), to avoid the part most affected by the Doppler effect. As we can see in Figure 5, the central part of the spectrogram shows curves, corresponding to frequency changes. Extracting line parameters is difficult in such case.
Method 7 Higher tolerance of local declinations. In this method, the range of possible declination of each line was increased, from 2 nearest neighbors of the average of the last 3 points, to the range of a musical tone, i.e., a semitone below and a semitone above. As a result, lines for higher frequencies were properly tracked.
Method 8 Modification of determining the gradient of each line, through searching for the most frequent gradient. In this method, when line tracking was completed, the mode of gradient values for ten-point segments was indicated as the new gradient.
Method 9 Variation of the method above: three most frequent values were found (i.e., the mode and the next two most frequent values), and their average was used as the new gradient value.

### 3.3. Classification

In this work, we decided to use SVM, random forests, and artificial neural networks as classifiers, as well as the time series based approach to classify shapelets. Random forests yielded good results in our previous works, so we considered this classifier to be a good choice. SVM and neural nets were also selected, in order to have a variety of classifiers for comparison, and also these classifiers are commonly used in similar research. We applied R [64] to use SVM and random forest classification, and WEKA [65] for neural networks. Since our data set is not big, we did not use deep learning, which requires a lot of data to be efficient; a multi-layer perceptron was applied. For time-series approach, fast shapelet algorithm was applied to calculate shapelets [62], to represent the evolution of each of our 85 basic features in time. This way we obtained a decision tree for each basic feature. Next, we selected the best trees to create a forest.

Random forest is a set of decision trees, built with minimizing bias and correlations between the trees. Each tree is built without pruning, for a different *N*-element bootstrap sample of the *N*-element training set. A bootstrap sample is obtained through drawing with replacement. For a *K*-element feature set, *k* features are randomly selected for each node of any tree. The best split on these *k* features is applied to split the data in the node. Gini impurity criterion is minimized to choose the split. The Gini criterion measures how often an element would be incorrectly labeled, if random labeling an object according to the distribution of labels in the subset is applied. Gini criterion is also applied to estimate the significance of features (and thus select best features in feature selection), as the mean decrease in Gini coefficient measures how each feature contributes to the homogeneity of the nodes and leaves of the trees.

To obtain the forest of *M* trees, the procedure described above is repeated *M* times. Classification is performed by simple voting of all trees. Standard settings in the randomForest package in R [64] were used in our research, i.e., with M = 500, and $k=\sqrt{K}$.

SVM classifiers look for a decision surface (hyperplane) maximizing the margin around the decision boundary. The data points that are closest to the hyperplane and are used to maximize the margin of the classifier are called support vectors. To achieve linear separation of data, SVM modifies the data space using a kernel function. We applied linear, quadratic, and RBF (radial basis function) kernels. Each kernel has parameters which must be tuned to achieve good performance. Grid search of these parameters for each kernel was performed using tune function in R.

Artificial neural network (ANN) is a computing system made up of a number of simple, highly interconnected processing elements, called neurons. These elements process information by their dynamic state response to external inputs. The structure of ANN can be very complex, and the more data, the better. In our experiments, with a relatively small data set, we decided to use multi-layer perceptron (MLP) with momentum equal to 0.9, implemented in WEKA [65]. The network had one hidden layer, with sigmoid activation function and the number of neurons equal to the average of the number of features and the training objects (default setting).

All these classifiers were run on the data represented by various feature sets, based on representations shown in Section 3.2.1, Section 3.2.2 and Section 3.2.4. We performed 10-fold cross validation CV-10, using 90% of the data for training and the remaining 10% of the data for testing. For random forests and SVM this procedure was repeated 10 times, and the results were averaged to estimate the accuracy of each classifier. For neural networks CV-10 was repeated only once, as it was more time consuming. The best classifiers were selected to the final classifier, as an ensemble classifier.

#### 3.3.1. Forest of Shapelets

Additionally, we applied fast shapelets algorithm for our data classification [62]. Shapelets allow classifying time series that contain an occurrence of the shapelet, within a previously learned distance threshold, based on trees. One tree corresponds to one audio feature in our case. The nodes contain shapelets (snippets of the time series corresponding to this feature) that allow differentiating between groups of objects, and thresholds for traversing the subtrees, directing into one of the children nodes (or leafs). Leafs represent particular classes: deceleration, acceleration, or stable speed.

Decision trees are constructed in order to match time series to shapelets. As mentioned above, each node of a tree consists of a pair: a shapelet, which represents a shape that was found in time series data, and a distance threshold value. If a new time series is classified, it is analyzed to find the part of the time series (a subsequence of the same length as shapelet in the node), which minimizes the distance between a part and the shapelet in the node. If the distance between the shapelet and the subsequence is less than the threshold, then time-series data are propagated to the left branch, otherwise to the right branch of the shapelet tree. Classification procedure ends in the tree leaf, to which a class label is assigned.

**Figure 7:**
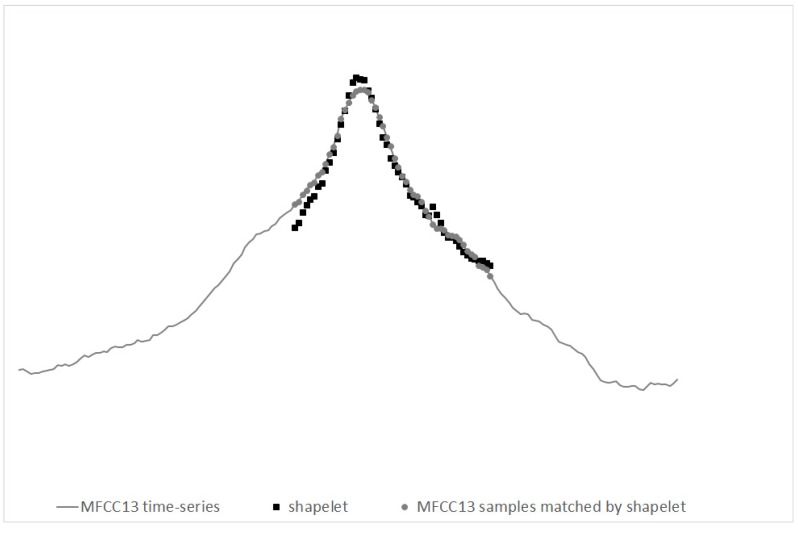
Exemplary shapelet for MFCC13, together with the part of MFCC13 evolution matching this shapelet best.

In Figure 7 we illustrate an exemplary shapelet, for the evolution of MFCC13 in time, shown together with the part of MFCC13 matching this shapelet best.

In our approach, we decided to construct a classifier consisting of a set of trees, where each tree is based on shapelet algorithm for one of 85 features, evolving in time. The classification is performed by simple voting of all trees in this forest. We chose the best performing features to this forest classifier. However, the results for this classifier were not among the best results from all classifiers. Therefore, we did not add this classifier to the final ensemble.

## 4. Results and Discussion

We started our experiments with the basic 85 features (Section 3.2.1), which yielded quite good results. We used the following classifiers: RF—random forest, SVML—SVM with linear kernel, SVMQ—SVM with quadratic kernel, SVMR—SVM with RBF kernel, and MLP. We obtained the following accuracy:

- RF: 90.5%,
- SVM: SVML 85.4%, SVMQ 87.1%, SVMR 90.9%,
- MLP: 88.6%.

The results obtained for lines (Section 3.2.4) are shown in Table 1. As we can see, the best results are obtained for lines calculated using Method9; good results were also obtained for Method4. RF performed better than SVM in these experiments.

**Figure 8:**
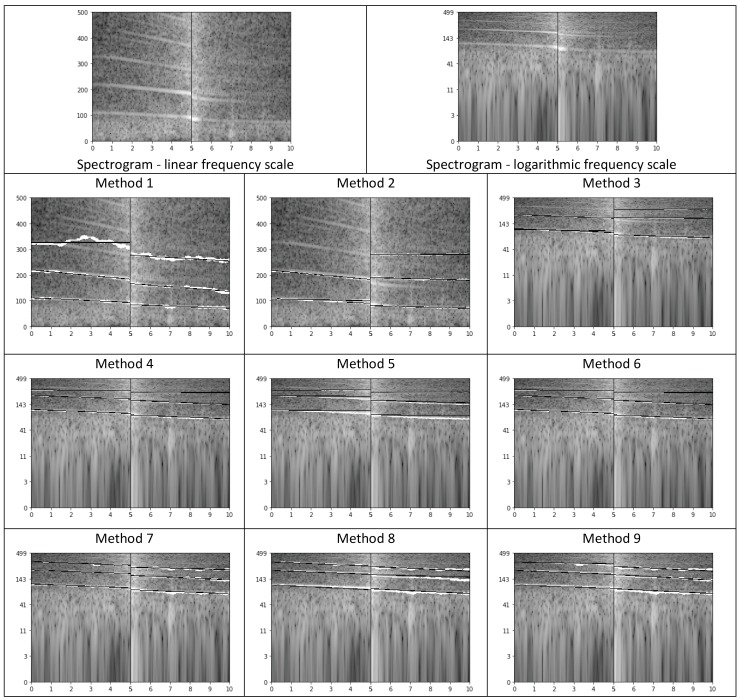
Spectrograms with paths (marked in white) and lines (black) generated by the evaluated methods (Section 3.2.4). We took deceleration as an example for illustration purposes. The horizontal axis represents time, the vertical axis represents frequency, and gray level represents the magnitude; the higher the luminance level, the higher the magnitude.

In Figure 8 we illustrate the difficulties encountered when calculating lines using the methods described in Section 3.2.4. As we can see in the original spectrogram, lines for the sound representing the car approaching the microphone are more clearly visible (for this example). In Method 1 the lines visible in the spectrogram are often lost, but in Method 2, where we use a high-resolution spectrogram, the paths better fit the lines. Methods 3–9 use spectrograms in a logarithmic frequency scale. The advantage of this solution is that in the logarithmic frequency scale the lines we search should be parallel. Therefore, we can easily detect a false line, which does not correspond to any target line in the spectrogram, as its slope will differ from the other two. It can be used to verify the found lines. However, the drawback of the logarithmic frequency scale is that the spectrogram is very blurred for very low frequencies, and our target lines are very close to each other. Additionally, they are weakly sloping, while the slope is the indicator of the type of speed change. Method 4 simplifies the procedure of searching candidates for the lines, and as we can see the found paths and lines are almost accurate in the exemplary sound. In Method 5 we use weighted linear regression and we get worse approximation results. The rest of our methods (6–9) give good results, comparable with Method 4, in the presented example.

We also ran MLP for the lines calculated using Method9, but the results were not encouraging, i.e., 76.6% only.

We also performed experiments on representations combining basic 85 features and lines. The obtained results are shown in Table 2.

The data representation using trends in feature evaluation in time (Section 3.2.2) yielded a high dimensional feature vector, and since we added basic 85 features (averages) to this representation, we obtained 595 features. We performed classification using this representation directly, but we also decided to perform smoothing of data before fitting power trends, and use feature selection. Smoothing was performed in three versions: with smoothing (averaging) over 3 points (SM3), 5 points (SM5), and seven points (SM7). Feature selection had 3 versions. Two of them (SEL1 and SEL2) were based on $R^{2}$ value; since $R^{2}$ is a measure of fitting the curve to the trend, these selection methods removed trend parameters incorrectly matched to features. The third selection method, SEL3, was based on the mean decrease of Gini coefficient in random forests. In SEL1 we selected only trend based features for which $R^{2}$ reached at least 0.7, whereas in SEL2 we left the features for which the median $R^{2}$ value for at least one class was higher than 0.7. The obtained results are shown in Table 3. As we can see, the results are better than for lines, which is not surprising, as in lines we only had 24 features, whereas here we had many more features. The best results were obtained for SVM with RBF kernel as classifier, for feature selection method SEL3, and for SEL2 with SM3 smoothing.

**Figure 9:**
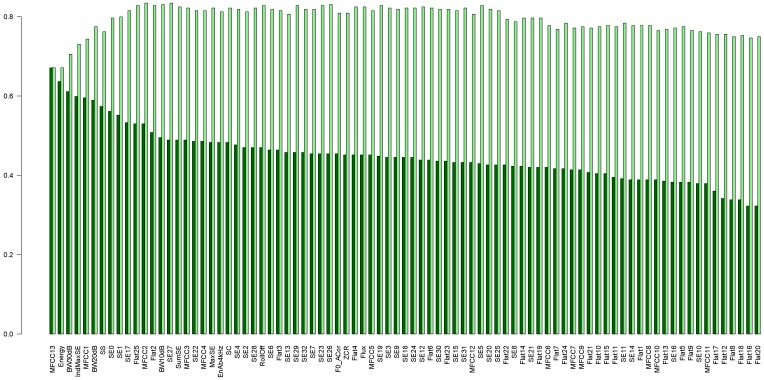
Accuracy obtained using shapelets. Features sorted according to the decreasing accuracy of shapelet trees. Dark-green bars represent accuracy for single trees, light-green bars represent the accuracy of the forest, created by adding trees one by one. Graph obtained for the left channel data.

In experiments with the forest of shapelets, we selected features yielding the best results to create the forest. Separate experiments were performed with the data representing the right and the left channel, and the list of the best features differed for these data. For the left channel we had: MFCC13, Energy, BW30dB, IndMaxSE, MFCC1, BW20dB, SS, SE0, SE1, SE17, Flat25, and MFCC2, as shown in Figure 9. The classifiers (trees) obtained for shapelets for these features constitute our forest. We added trees to the forest one by one, starting with the best feature (MFCC13, corresponding to Energy), and then added the next ones in the order of decreasing accuracy. We stopped adding trees after the accuracy of the forest started to drop. Therefore, our forest consists of 12 trees. This forest yielded the accuracy of about 84%.

The classifiers which achieved the highest accuracy were selected to the final ensemble classifier. We chose five RF models, based on the following representations: lines calculated using Method9, basic 85 features, combined basic 85 features and lines calculated using Method4, combined basic 85 features and lines calculated using Method9, and trends with smoothing over three points with SEL2. Also five SVMR models were chosen, calculated for following feature sets: basic 85 features, combined basic 85 features and lines calculated using Method4, combined basic 85 features and lines calculated using Method9, trends with smoothing over three points with SEL2, and SEL3 on trends (without smoothing). These ten classifiers formed the ensemble of classifiers, and the classification using this ensemble was performed through simple voting. Accuracy of the ensemble was 94.7%. The confusion matrix for both stereo channels together for the ensemble classifier is shown in Table 4.

As we can see, this classifier never mistakes acceleration and deceleration, and in only 16 out of 188 cases (8.5%) stable speed was classified as speed change. Therefore, unjust classification of proper driving is avoided as much as possible.

Since we obtained high accuracy for MLP classifier built for the combined basic 85 features and lines calculated Method4, we tried to add this classifier to the ensemble, to have 11 classifiers in it. However, after adding this particular classifier to our ensemble the overall accuracy was a bit lower, namely decreased to 94%.

## 5. Conclusions

In the presented work, we proposed the methodology for detecting speed changes based on audio data, recorded for vehicles passing the audio recorder. Each recording represents a single drive of one car in order to avoid interfering sounds at this stage of the work. The presented results show that even a simple feature vector, representing lines in a spectrogram, yields 90% accuracy, even though tracking these lines is difficult, and errors in these parameters are possible. The ensemble classifier yields the best results, better than single classifiers composing it, and an encouraging accuracy of almost 95% can be achieved this way. What is especially important, accelerating was not mistaken with decelerating and vice versa. Detecting speed changes from audio data does not require accessing data from sensors installed in cars, and can be performed in any weather (assuming that the recorder is protected), or at low visibility conditions. The outcomes of this work can help assessing how the drivers react near speed cameras, and become a basis for campaigns aiming at the improvement of traffic safety. As future work, we are planning to acquire more data, and also work with recordings representing multiple vehicles passing the measurement point.

## Figures and Tables

**Table 1:** Classification accuracy for speed changes for 24 features describing lines, depending on the method of determining the line. The three best results are shown in bold.

Method	RF	SVML	SVMQ	SVMR
Method1	84.9%	72.9%	74.1%	74.5%
Method2	80.4%	74.1%	75.9%	77.6%
Method3	80.7%	75.3%	75.6%	77.8%
Method4	**89.8%**	**89.4%**	89.3%	89.0%
Method5	81.3%	75.0%	76.6%	78.5%
Method6	81.7%	74.6%	76.1%	80.3%
Method7	87.3%	82.6%	84.2%	88.3%
Method8	83.1%	76.5%	77.8%	81.2%
Method9	**90.3%**	85.6%	87.4%	88.3%

**Table 2:** Classification accuracy for speed changes for combined feature vectors: 85 basic features and lines, calculated using 2 best performing methods. The three best results are shown in bold.

Method	RF	SVML	SVMQ	SVMR	MLP
Basic 85 + Method4	**92.6%**	89.7%	89.8%	91.8%	**92.6%**
Basic 85 + Method9	**91.8%**	86.2%	86.5%	91.5%	89.7%

**Table 3:** Classification accuracy for speed changes for feature vectors describing trends, depending on feature selection and smoothing. NS: no smoothing. The two best results are shown in bold.

Method	Features	RF	SVML	SVMQ	SVMR
NS, no selection	595	85.6%	87.5%	88.2%	90.2%
SM3, no selection	595	85.7%	87.1%	87.8%	88.9%
SM5, no selection	595	85.9%	86.1%	87.1%	89.9%
SM7, no selection	595	86.3%	86.4%	87.3%	90.1%
No smoothing, SEL1	217	88.4%	84.7%	86.7%	88.6%
SM3, SEL1	283	88.5%	85.3%	86.8%	89.7%
SM5, SEL1	340	87.8%	82.7%	85.1%	89.2%
SM7, SEL1	364	87.4%	85.3%	87.0%	89.6%
No smoothing, SEL2	91	90.8%	85.7%	88.3%	90.7%
SM3, SEL2	97	90.8%	85.1%	88.2%	**91.0%**
SM5, SEL2	100	90.6%	84.5%	87.1%	90.6%
SM7, SEL2	106	90.6%	84.5%	86.9%	90.5%
SEL3	345	86.4%	88.2%	89.2%	**91.3%**

**Table 4:** Confusion matrix for the ensemble classifier.

Classified as:	Acceleration	Deceleration	Stable Speed
Acceleration	216	0	6
Deceleration	0	216	12
Stable speed	5	11	172
